# OX40/OX40L Inhibitors in Atopic Dermatitis: Current Evidence on Short-term Safety

**DOI:** 10.2340/actadv.v106.adv-2026-0665

**Published:** 2026-10-12

**Authors:** Kian Karimian, Daniel Isufi, Mikkel Bak Jensen, Raj Chovatiya, Christopher G. Bunick, Tiago Torres, Jakob Ferløv Baselius Schwensen, Nikolai Loft

**Affiliations:** 1 National Allergy Research Centre, Department of Dermatology and Allergy, Herlev and Gentofte Hospital, Herlev, Denmark; 2 Department of Dermatology and Allergy, Herlev and Gentofte Hospital, University of Copenhagen, Herlev, Denmark; 3 Copenhagen Research Group for Inflammatory Skin, Herlev and Gentofte Hospital, Hellerup, Denmark; 4 Institute of Clinical Medicine, Faculty of Health and Medical Sciences, University of Copenhagen, Copenhagen, Denmark; 5 Department of Medicine, Chicago Medical School, Rosalind Franklin University of Medicine and Science, North Chicago, IL, United States; 6 Center for Medical Dermatology and Immunology Research, Chicago, IL, USA, United States; 7 Department of Dermatology, Yale School of Medicine, New Haven, CT, United States; 8 Program in Translational Biomedicine, Yale School of Medicine, New Haven, CT, United States; 9 Department of Dermatology, Centro Académico Clínico ICBAS/Santo António, Porto, Portugal; 10 Instituto de Ciências Biomédicas Abel Salazar, University of Porto, Porto, Portugal

The OX40 pathway has been targeted by multiple investigational advanced therapies for atopic dermatitis (AD), showing promising efficacy with the potential for disease modification over time (1–7). These treatments target either the OX40 receptor directly on activated T-cells (rocatinlimab and telazorlimab) or the OX40 ligand (OX40L) expressed on antigen-presenting cells (amlitelimab) to reduce T-cell activity and rebalance T-cell-mediated immune dysregulation. As these novel therapies approach clinical application, understanding their short-term safety is important. Thus, this study evaluates the safety of OX40/OX40L inhibitors in published randomized controlled trials (RCTs) in patients with AD.

## MATERIALS AND METHODS

PubMed, Embase and Web of Science were systematically searched using search terms (Table SI) for OX40 treatments in AD (PROSPERO; CRD420251171265). Seven studies including 1 conference abstract (*n*=3,501 patients) were included for final analysis (1–7) (**Fig. 1**, Tables SII-SIII).

**Fig. 1.**
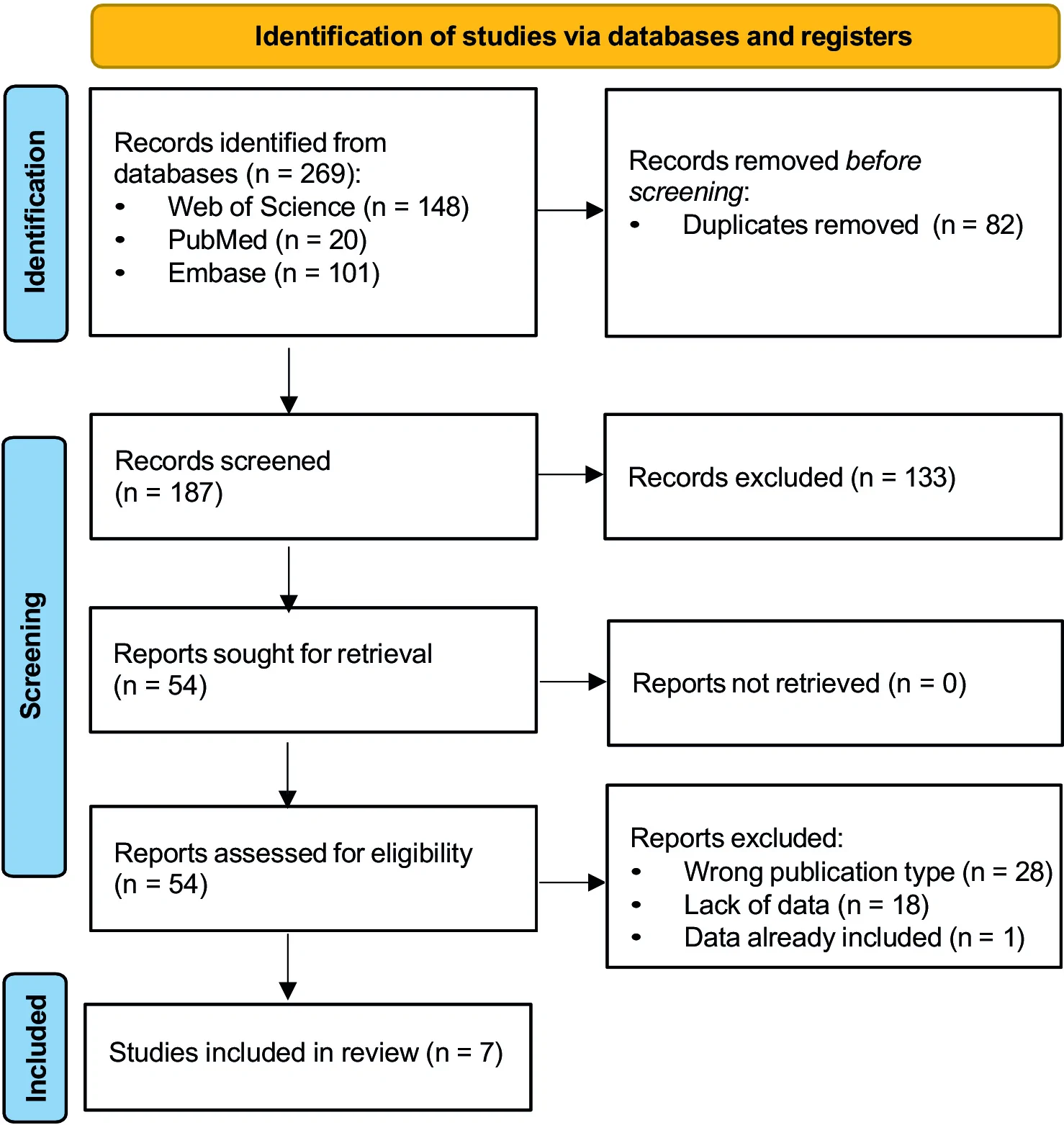
Flowchart of study selection

Risk of bias was assessed using Cochrane RoB 2 (Table SIV). Due to significant clinical and methodological heterogeneity across the included trials, including variations in drug targets, study phases, treatment durations, baseline clinical characteristics and the use of concomitant therapies, a pooled quantitative meta-analysis was not performed. Since data were not pooled, formal statistical heterogeneity testing (e.g. I^2^ statistics) was not applicable. Consequently, the findings are presented as a systematic review (Table SV).

## RESULTS

### Amlitelimab

Data for amlitelimab were reported in 1 phase 2a (2) and 1 phase 2b trial (4) (*n*=370 patients). The proportions of patients experiencing any AEs were 54.2% (2) (69.0% for placebo) and 66.9% (4) (60.3% for placebo). The most frequently reported AEs were nasopharyngitis (8.5% (2) and 10.9% (4)), headache (5.1% (2) and 6.1% (4)), upper respiratory tract infection (5.1% (2)) and pyrexia (3.4% (2)). The rates of serious adverse events (SAEs) were 1.7% (2) and 2.6% (4). Reported SAEs included an infected dermal cyst (*n*=1 (2)). The discontinuation rate due to AEs was 4.5% (4). These included bullous dermatitis and elevated liver enzymes (4).

### Rocatinlimab

Data for rocatinlimab were reported in 1 phase 2b (1) and 3 phase 3 trials (6, 7) (*n*=1,816 patients). The proportions of patients experiencing any AEs ranged from 68.2% to 81.0% (1, 6, 7) (63.3% to 71.9% for placebo). The most frequent AEs were pyrexia (10.3% to 16.7% (1, 6)), nasopharyngitis (8.8% to 13.9% (1, 6)), headache (7.2% to 8.8% (1, 6)) and chills (3.7% to 11.1% (1, 6)). Pyrexia events were generally mild to moderate, occurred predominantly after the initial dose and did not result in discontinuation (1, 6). SAEs occurred in 1.8% (6), 3.7% (1), 3.7% (7) and 3.9% (6) of patients. Reported SAEs included musculoskeletal events (*n*=5 (6)), gastrointestinal ulceration (*n*=3 (6)), appendicitis (*n*=2 (6)), cellulitis (*n*=2 (6)) and anaphylactic reactions (*n*=2 (6)). Discontinuation due to AEs occurred in 1.4% (7), 2.0% (6), 2.6% (6) and 8.8% (1) of patients. These included exacerbation of atopic dermatitis (6), aphthous ulcers (6), headache (6), ileal ulcer (6) and anal abscess (1).

### Telazorlimab

Data for telazorlimab were reported in 1 phase 2a (5) and 1 phase 2b trial (3) (*n*=352 patients). The overall rates of AEs were 63.0% (5) (62.5% for placebo) and 66.0% (3) (60.4% for placebo). The most common AEs observed were nasopharyngitis (8.2% (3) and 8.7% (5)), upper respiratory tract infection (6.9% (3) and 8.7% (5)), and headache (5.9% (3) and 13.0% (5)). Pyrexia was not registered in any of the studies. SAEs were reported in 2.6% (3) of patients. Reported SAEs included viral infection (*n*=1 (3)), exacerbations of AD (*n*=4 (3)), fatal hypertension (*n*=1 (3)) and coronary artery occlusion (*n*=1 (5)). The fatal hypertension, coronary artery occlusion and 2 of the AD exacerbations were not considered treatment related. The discontinuation rate due to AEs was 1.3% (3), and these included paresthesia, pernicious anaemia and thrombocytosis (3).

## DISCUSSION

Due to the clinical and methodological heterogeneity among trials, direct comparisons of these therapies must be interpreted with caution. AEs were generally higher for active arms compared to placebo, except1 rocatinlimab phase 3 study which reported comparable proportions (69.8% vs 69.3%) (6). Notably, pyrexia patterns differed by drug design. Rocatinlimab, an afucosylated IgG1 antibody enhanced for antibody-dependent cellular cytotoxicity (ADCC) showed a higher reported occurrence of pyrexia. In contrast, telazorlimab (nonafucosylated IgG1) showed no pyrexia, and amlitelimab (nondepleting IgG4) showed lower rates. This suggests that rapid cell depletion driven by enhanced ADCC may be related to this AE rather than OX40 receptor binding or standard IgG1 activity. However, further investigation is required. While most studies reported higher SAE rates in active arms, two rocatinlimab phase 3 trials (6) found lower rates (1.8% and 3.9%) compared to placebo (4.4% and 5.5%).

Despite reassuring early trial data, post-trial monitoring revealed imbalances in infection and Kaposi sarcoma occurrences, prompting Kyowa Kirin to discontinue rocatinlimab development (8). The development of Kaposi sarcoma, driven by human herpesvirus 8 (HHV-8), suggests that prolonged OX40 blockade may impair T-cell-mediated immunosurveillance, which is necessary for controlling latent viral infections. These safety signals necessitate a reevaluation of the balance between targeted immunomodulation and broader immunosuppression with OX40/OX40L blockade. Furthermore, Sanofi recently discontinued the clinical development of amlitelimab for AD, concluding that the totality of efficacy and safety evidence did not represent a meaningful improvement over the current standard of care (9). This highlights the potential need for pretreatment screening and vaccination protocols. Future analyses must prioritize characterizing the specific clinical context of SAEs.

This study has several limitations, primarily the small number of trials and short evaluation periods (12 to 24 weeks), which precluded long-term safety assessment. Additionally, phase 3 data were available only for rocatinlimab, whereas amlitelimab and telazorlimab were restricted to phase 2 trials, limiting comparability across treatments. Furthermore, although a conference abstract provides less comprehensive data that have not undergone full peer review, its inclusion was deemed necessary to capture the most up-to-date Phase 3 safety landscape. Importantly, the data originate from a randomized controlled trial that is governed by strict ICH GCP reporting standards. This ensures that the findings remain relevant for understanding the current safety profile.

Although there were more AEs in the active arms, most were mild. However, severe safety signals must be addressed, particularly Kaposi sarcoma, which halted the development of rocatinlimab.

## Data Availability

The data that support the findings of this study are available from the corresponding author upon reasonable request.
