# Supplementary material for: OX40/OX40L Inhibitors in Atopic Dermatitis: Current Evidence on Short-term Safety
Source: Acta Derm Venereol. 2026 Oct 12;106:0665. doi: 10.2340/actadv.v106.adv-2026-0665 (PMC13656841; doi:10.2340/actadv.v106.adv-2026-0665)
Supplement: Supplementary file 1 [file ActaDv-106-0665-s0001.pdf]

SUPPLEMENTAL TABLE LEGENDS

Table SI. Search strategy conducted the 18th of October 2025

("Amlitelimab" OR "KY1005" OR "SAR445229")<br>*OR*<br>*("Rocatinlimab" OR "AMG 451" OR "KHK4083")*<br>*OR*<br>*("Telazorlimab" OR "ISB 830" OR "GBR 830" OR "GBR-830")*<br>*AND*<br>*("phase 2" OR "phase II" OR "phase 3" OR "phase III")*<br>*AND*<br>*("atopic dermatitis" OR "atopic eczema")*

Table SII. General characteristics of the OX40/OX40L inhibitors

Drug	Antibody design	Main target	Affinity
Amlitelimab	Fully human, IgG4 monoclonal (Non-depleting)	OX40L	0.07 nM[S1]
Rocatinlimab	Fully human, afucosylated IgG1 monoclonal (Enhanced ADCC)	OX40	3.00 nM[S2]
Telazorlimab	Humanized IgG1 monoclonal (ADCC)	OX40	59.00 nM[S2]

ADCC: Antibody-Dependent Cellular Cytotoxicity; nM: Nanomolar

Table SIII. General characteristics of the included studies

OX40/OX40L	Trial	Phase	Drug dose	Concomitant treatment	Evaluation time point [weeks]	OX40/OX40L patients [n]	OX40/OX40L males [n]	OX40/OX40L age [mean ± SD]	Placebo patients [n]	Placebo males [n]	Placebo age [mean ± SD]
	(NCT03754309)[2]	2a	200 mg, 500 mg	No	16	59	32	34.35 ± 12.30	29	19	32.10 ± 10.80
Amlitelimab (OX40L)	STREAM-AD[4] (NCT05131477)	2b	250 mg Q4W + LD, 250 mg Q4W, 125 mg Q4W, 62.5 mg Q4W	Topical rescue therapy	24	311	170	38.15 ± 14.63	78	49	36.40 ± 13.10
Rocatinlimab (OX40)	(NCT03703102)[1]	2b	150 mg Q4W, 600 mg Q4W, 300 mg Q2W, 600	No	18	216	127	37.70 ± 14.78	57	31	38.70 ± 14.40

			mg Q2W								
	ROCKET-HORIZON[6] (NCT05651711)	3	300 mg	Rescue therapy	24	544	295	37.80 ± 14.60	180	102	40.40 ± 15.60
	ROCKET-IGNITE[6] (NCT05398445)	3	150 mg, 300 mg	Rescue therapy	24	540	325	38.50 ± 15.40	218	122	37.10 ± 14.00
	ROCKET-SHUTTLE[7] (NCT05724199)	3	150 mg, 300 mg	Mild to medium steroids and TCI	24	516	308	38.80 ± 14.80	230	135	37.50 ± 14.70
Telazorlimab (OX40)	(NCT02683928)[5]	2a	10 mg/kg	Emollients and mild steroids	12	46	21	36.20 ± 13.40	16	11	40.40 ± 15.10
	(NCT03568162)[3]	2b	300 mg Q2W, 600 mg Q2W, 75 mg Q4W, 300 mg Q4W	No	16	306	152	38.28 ± 14.53	154	63	36.15 ± 13.45

LD: Loading Dose; N: Number; Q2W: Every 2 weeks; Q4W: Every 4 weeks; TCI: Topical Calcineurin Inhibitors

Table SIV. Risk of Bias Assessment

Study	D1: Randomization Process	D2: Deviations from Intended Interventions	D3: Missing Outcome Data	D4: Measurement of the Outcome	D5: Selection of the Reported Result	Overall Risk
Guttman-Yassky (2019)[5]	Low	Low	Some concerns	Low	Low	Some concerns
Guttman-Yassky (2023)[1]	Low	Low	Some concerns	Low	Low	Some concerns
Guttman-Yassky (2025)[6]	Low	Low	Low	Low	Low	Low
Rewerska (2024)[3]	Low	Low	Low	Low	Low	Low
Simpson (2025)[7]	High	High	High	Some concerns	High	High
Weidinger (2023)[2]	Low	Low	Low	Low	Low	Low
Weidinger (2025)[4]	Low	Low	Low	Low	Low	Low

Table SV. Adverse events for OX40/OX40L and placebo

OX40/OX40L	Trial	Drug	Treatment period [weeks]	Patients [n]	Any AEs [n]	Serious AEs [n]	Disc due to AEs [n]	Aphthous ulcer [n]	Exacerbation of atopic dermatitis [n]	Chills [n]	COVID-19 [n]	Fatigue [n]	GI-disorders [n]	Headache [n]	Nasopharyngitis [n]	Pyrexia [n]	Upper respiratory tract infection [n]
Amlitelimab (OX40L)	(NCT03754309)[2]	Amlitelimab	16	59	32 (54.2%)	1 (1.7%)	NA	NA	7 (11.9%)	NA	NA	NA	1 (1.7%)	3 (5.1%)	5 (8.5%)	2 (3.4%)	3 (5.1%)
		Placebo		29	20 (69.0%)	0 (0.0%)	NA	NA	9 (31.0%)	NA	NA	NA	3 (10.3%)	1 (3.4%)	6 (20.7%)	0 (0.0%)	1 (3.4%)
	STREAM-AD[4] (NCT05131477)	Amlitelimab	24	311	208 (66.9%)	8 (2.6%)	14 (4.5%)	NA	NA	NA	24 (7.7%)	NA	NA	19 (6.1%)	34 (10.9%)	NA	NA
		Placebo		78	47 (60.3%)	1 (1.3%)	5 (6.4%)	NA	NA	NA	5 (6.4%)	NA	NA	2 (2.6%)	7 (9.0%)	NA	NA
Rocatinlimab (OX40)	(NCT03703102)[1]	Rocatinlimab	18	216	175 (81.0%)	8 (3.7%)	19 (8.8%)	15 (6.9%)	NA	24 (11.1%)	NA	NA	NA	19 (8.8%)	30 (13.9%)	36 (16.7%)	NA
		Placebo		57	41 (71.9%)	1 (1.8%)	12 (21.1%)	0 (0.0%)	NA	0 (0.0%)	NA	NA	NA	1 (1.8%)	9 (15.8%)	2 (3.5%)	NA
	ROCKET-HORIZON[6] (NCT05651711)	Rocatinlimab	24	544	371 (68.2%)	10 (1.8%)	14 (2.6%)	22 (4.0%)	104 (19.1%)	33 (6.1%)	22 (4.0%)	NA	NA	39 (7.2%)	48 (8.8%)	56 (10.3%)	34 (6.3%)
		Placebo		180	114 (63.3%)	8 (4.4%)	5 (2.8%)	1 (0.6%)	48 (26.7%)	2 (1.1%)	2 (1.1%)	NA	NA	7 (3.9%)	21 (11.7%)	2 (1.1%)	6 (3.3%)
	ROCKET-IGNITE[6] (NCT05398445)	Rocatinlimab	24	540	377 (69.8%)	21 (3.9%)	11 (2.0%)	22 (4.1%)	60 (11.1%)	20 (3.7%)	18 (3.3%)	20 (3.7%)	NA	46 (8.5%)	56 (10.4%)	75 (13.9%)	25 (4.6%)
		Placebo		218	151 (69.3%)	12 (5.5%)	2 (0.9%)	2 (0.9%)	56 (25.7%)	4 (1.8%)	10 (4.6%)	8 (3.7%)	NA	10 (4.6%)	19 (8.7%)	10 (4.6%)	9 (4.1%)
		Rocatinlimab	24	516	369	19	7	NA	NA	NA	NA	NA	NA	NA	NA	NA	NA

	ROCKET-SHUTTLE[7] (NCT05724199)				(71.5%)	(3.7%)	(1.4%)										
		Placebo		230	155 (67.4%)	2 (0.9%)	1 (0.4%)	NA	NA	NA	NA	NA	NA	NA	NA	NA	NA
Telazorlimab (OX40)	(NCT02683928)[5]	Telazorlimab	12	46	29 (63.0%)	NA	NA	NA	6 (13.0%)	NA	NA	2 (4.3%)	0 (0.0%)	6 (13.0%)	4 (8.7%)	NA	4 (8.7%)
		Placebo		16	10 (62.5%)	NA	NA	NA	2 (12.5%)	NA	NA	1 (6.3%)	2 (12.5%)	4 (25.0%)	2 (12.5%)	NA	2 (12.5%)
	(NCT03568162)[3]	Telazorlimab	16	306	202 (66.0%)	8 (2.6%)	4 (1.3%)	NA	63 (20.6%)	NA	NA	6 (2.0%)	NA	18 (5.9%)	25 (8.2%)	NA	21 (6.9%)
		Placebo		154	93 (60.4%)	2 (1.3%)	3 (1.9%)	NA	29 (18.8%)	NA	NA	1 (0.6%)	NA	7 (4.5%)	14 (9.1%)	NA	12 (7.8%)

AEs: Adverse Events; GI: Gastrointestinal; N: Number; NA: Not Available

SUPPLEMENTAL REFERENCES

S1. Wickman G, Kang BH, Meinke J, Raso S, Oh J, Shaheen H, et al. Preclinical potency, affinity, and pharmacokinetics of APG990, a half-life extended antibody against OX40L. Poster presented at: 2025 Winter Clinical Dermatology Conference; 2025 Feb 14-19; Hawaii, USA.

S2. Biris N, Macoin J, Carretero L, Perez C, Zhao C. Development and characterization of STAR-0310: a novel OX40 antagonistic monoclonal antibody. Poster presented at: European Academy of Allergy and Clinical Immunology (EAACI) Congress 2024; 2024 May 31 - Jun 3; Valencia, Spain.
